# Deliberate Self-Poisoning Presenting to an Emergency Medicine Network in South-East Melbourne: A Descriptive Study

**DOI:** 10.1155/2014/461841

**Published:** 2014-06-12

**Authors:** Asheq Rahman, Catherine Martin, Andis Graudins, Rose Chapman

**Affiliations:** ^1^Monash Health, 135 David Street, Dandenong, VIC 3175, Australia; ^2^Australian Catholic University, 115 Victoria Parade, Fitzroy, VIC 3065, Australia; ^3^Monash University, Clayton, VIC 3168, Australia

## Abstract

*Background*. Deliberate self-poisoning (DSP) comprises a small but significant proportion of presentations to the emergency department (ED). However, the prevalence and patient characteristics of self-poisoning attendances to EDs in Victoria have not been recently characterised. *Aim*. To identify and compare the characteristics of adult patients presenting to the three EDs of Monash Health following DSP. *Methods*. Retrospective clinical audit of adult DSP attendances between 1st July 2009 and 30th June 2012. *Results*. A total of 3558 cases over three years were identified fulfilling the search criteria. The mean age of patients was 36.3 years with the largest numbers aged between 18 and 30 (38%). About 30% of patients were born overseas. Forty-eight percent were discharged home, 15% were admitted to ED short stay units, and 5% required ICU admission. The median ED length of stay was 359 minutes (IQR 231–607). The most frequently reported substances in DSP were benzodiazepines (36.6%), paracetamol (22.2%), and antipsychotics (12.1%). Exposure to more than one substance for the episode of DSP was common (47%). *Conclusion*. This information may help identify the trends in poisoning substances used for DSP in Victoria, which in turn may provide clinicians with information to provide more focused and targeted interventions.

## 1. Introduction

Deliberate self-poisoning (DSP) variably accounts for 0.5 to 2% of all admissions to Emergency Departments (EDs) [1, 2]. For the purpose of this paper the term DSP was used to describe those patients who had intentionally ingested substances to cause themselves harm. Patients may have associated complex medical and mental health problems and the DSP episodes may result in a high degree of resource use and consequently cost to the health service and the community [3]. The ED is usually the initial point of contact for individuals presenting with deliberate self-harm [3–6]. In 1999, a report on adult poisoning in Victoria indicated that 4.9% of ED admissions were due to poisoning and medications were used in more than 50% of cases [2]. Although the societal burden of these admissions has been acknowledged internationally, little recent data exists on the prevalence and characteristics of patients presenting with self-poisoning to Victorian Emergency Departments.

There are very few recent Australian studies investigating the prevalence and patient characteristics of individuals presenting to the ED with DSP. Buykx et al. found that the majority of overdose patients were female in their 30s and referred for psychosocial assessment [7]. This is similar to previous studies of DSP patients. In a Victorian retrospective study from the late 1990s, Taylor et al. found that the majority of DSP presenters to their community teaching hospital were also women [1]. Single drugs such as paracetamol and antipsychotic agents were more commonly used in the repeat presenters. In another Australian retrospective study of 325 patients presenting with DSP in Queensland, the most common substances taken were benzodiazepines (39.5%), antidepressants (11.7%), or paracetamol (7.2%) [8]. Also in the mid-1990s, Buckley et al. reported that benzodiazepines, alcohol, and paracetamol figured highly in the most common substances ingested. Tricyclic antidepressant (TCA) poisoning was also common and responsible for a substantial proportion of DSP-related deaths in this study [9]. However, more recently, the same authors noted that there was a swing away from the prescribing of TCAs and a greater use of SSRI antidepressants resulting in less DSP morbidity and mortality [10]. As a result, over time, the epidemiology of substances implicated in DSP has changed and appears to parallel the marketing and frequency of prescribing of newer medications. In general, these previous studies describe more recent DSP characteristics in Queensland and New South Wales. There also have been some recent studies in Victoria examining how medications that are used to self-poison are acquired [11] and reporting on specific drugs like antidepressants [12]. However, detailed reports on DSP from Victoria are more dated and are as much as 15 to 25 years old. It is highly likely that, with the change in prescribing practice, newer prescription medications have taken the place of older drugs as preferred poisoning agents. As a result, the degree of toxicity, morbidity, and mortality may also have changed over the years.

This current study aims to describe the epidemiology of deliberate self-poisoning in adult patients presenting to the emergency departments of Monash Health, an urban health care network in South Eastern Melbourne.

## 2. Methods

### 2.1. Setting

Monash Health is one of the largest health care providers in Victoria. There are three acute hospitals with four EDs: one adult, one tertiary paediatric (Monash Medical Centre), and two mixed EDs (Dandenong and Casey Hospitals) with a total of around 171000 attendances/year. The catchment population of these three hospital areas is socioeconomically different [13].

### 2.2. Data Collection

For the purpose of this retrospective descriptive study data, all patients presenting with a triage diagnosis of overdose/poisoning/self-harm were identified in Symphony (Emergency Department clinical data system, Version 2.29, Ascribe plc, Bolton, UK) and reviewed for the presence of deliberate self-poisoning as a reason for presentation to the ED.

The extraction and recoding of the data was done in two phases. In the first phase, key words from the Triage Nurse's comments were used in Stata Statistical Software (Release 12 College Station, TX; StataCorp) to extract cases with DSP and remove cases of accidental, recreational poisoning, or drink-spiking poisoning. Manual exclusion of non-DSP cases was conducted following this and the final number of cases was 3558. In the second phase, the coding/recoding of the major classes of drugs/poisons was done according to the generic names and the codes were confirmed by using a web based free medical information system (NPS, http://www.nps.org.au/). Substances were categorized by class and any remaining drugs were grouped as others. Available patient demography, such as age, sex, marital status, country of birth, and location of the residence were included in the analysis. SEIFA (Socio Economic Indexes for Areas) Indexes rank areas in Australia according to relative socioeconomic advantage and disadvantage and an area with a lower SEIFA value indicates relatively greater disadvantage and a lack of advantage in general [14]. It might be noteworthy to mention that an area with a SEIFA value less than 1000 indicates lower relative socioeconomic advantage/disadvantage compared to other areas.

SEIFA Indexes (in particular the Index of Relative Socioeconomic Advantage and Disadvantage, IRSAD) were obtained from Australian Bureau of Statistics to assess the presence of any socioeconomic differences between the populations presenting to the three emergency departments (http://www.abs.gov.au/websitedbs/censushome.nsf/4a256353001af3ed4b2562bb00121564/seifa).

Most of the patients were from surrounding suburbs and postcodes were tabulated against frequency of patients coming from specific postcodes. As the addresses of patients were removed for the deidentification purpose, postcodes were the only locator and the Local Government Area (LGA) SIEFA scores have been used against the respective postcodes.

Relevant ED patient disposition information was obtained from electronically generated Monash Health ED Executive Report (Datasource: Health Central-Symphony) [15].

### 2.3. Data Analysis

Using Stata, descriptive statistics were generated. Differences in the occurrence and characteristics of self-poisoning presentations based on patient characteristics were assessed using different tests of significance, namely, Pearson's chi square test for comparing frequencies, ANOVA for comparing means (including application of Bonferroni, Scheffe, and Sidak test of comparison means between variables), and Kruskal Wallis for comparing median values. Statistical significance was considered at *α* < 0.05.

### 2.4. Inclusion Criteria

All patients were 18 years and over presenting to the Emergency Departments of the health service with deliberate self-poisoning between the 1st July 2009 and 30th June 2012.

### 2.5. Ethical Approval

Ethical approval was sought and gained from The Australian Catholic University and the Monash Health Human Research and Ethics Committee prior to commencement of data collection.

## 3. Results

### 3.1. Demography

During the study period, there were 512,282 presentations to Monash Health ED. There were 10,548 patients presenting with poisoning of which 3558 were classified as DSP in patients >18 years. This represented 0.7% of all ED presentations. The mean age of the patients was 36.3 years and 64.6% were female. The largest numbers of patients presenting with DSP were aged between 18 and 30 (38% of the total cases) (Table 1). Both in total, and in individual groups, females were predominant. This was especially noteworthy in the 18–30 years age group where 70% were female. Around 58% of DSP presenters were single and 30% were married. Significant differences in the number of females were observed in 18–30 years and 31–40 years age groups between the hospital areas; similar differences were observed for the males (Table 1).

The ethnic distribution of the DSP population indicated that patients who were born in Australia represented the greatest percentage of patients presenting with DSP (about 72%). However, it was not possible to identify the Aboriginal and Torres Islanders from those cases due to lack of data. Significant differences were found between hospital areas for the population who were not born in Australia. For example, Dandenong Hospital had a greater number of admissions of overseas born patients (Table 1). For patients born in countries other than Australia, England, India, New Zealand, Sri Lanka, Vietnam, and Afghanistan were the predominant countries of birth for cases with DSP. Significant main differences were found in the frequency of DSP cases for single, divorced/separated, and inadequately described groups among different hospital areas (Table 1).

In a separate analysis, we examined the postcode locations and SEIFA scores of the suburbs from which most of the patients originated; in majority, the plotted scores against postcodes for Casey and Dandenong hospitals were below 1000 and for Monash Medical Centre Clayton were above 1000. The average SEIFA score for Casey, Dandenong, and Monash Medical Centre Clayton was 984.38 (SD 32.97), 981.119 (SD 48.53), and 1043.09 (SD 59.97), respectively. The mean SEIFA scores between hospital areas were significantly different (*P* = 0.0002, one-way ANOVA). An intercomparison of mean SEIFA score (by using Bonferroni, Scheffe, and Sidak) for the three hospitals revealed that there were significant differences between Dandenong Hospital and Monash Medical Centre Clayton (Bonferroni, Scheffe, and Sidak, *P* = 0.001) and Casey hospital and Monash Medical Centre Clayton (Bonferroni and Sidak, *P* = 0.002, and Scheffe, *P* = 0.003). SEIFA scores for Dandenong and Casey areas were not different.

Around 47% of DSP patients presenting to ED were discharged home directly from the ED. This compares with 60% of patients being discharged home from the ED for all other reasons. Around 40% of the remaining DSP cases were admitted to either an ED short stay, medical, or mental health bed (Table 2). Seven percent of DSP patients were admitted to a mental health bed from the ED. A notable proportion of DSP cases (3.9%) left the ED before treatment was started. This compares to 4.7% of patients leaving prior to completion of treatment for all ED presentations [15]. There were significant differences in frequency of the major ED disposition categories between the hospital areas (Table 2). For example, more than 61% of the DSP cases remained in the same hospital campus for Dandenong, for the other two hospitals this was almost half.

Regarding the presentation of the DSP cases in the ED, no significant differences in the day or time were found between the Hospitals. However, about 50% of presentations to the ED were between 16.00 and 23.59 hours at all three sites.

The median length of stay in the Emergency Department (excluding time in the Short Stay Unit) was 359.5 minutes (IQR 231-607). Comparison of individual EDs revealed that the median length of stay was significantly longer at Monash Medical Centre compared to the other two EDs (Table 3). Around 15% of DSP patients (550 cases) were admitted to the ED Short Stay Units (SSU) of the three hospitals for longer observation periods. The median length of stay in SSU was 376.5 minutes (IQR 182-712) and was significantly different between the three hospitals. As a result, total length of stay for SSU admitted patients was 736 minutes (approximately 12.6 hours).

The most commonly reported class of drug involved poisoning was the benzodiazepines (36.6%), followed by paracetamol products (22.2%) and antipsychotics (12.2%). These three classes of drugs were reported in 71% of DSP cases (Table 4). Other common classes reported were the selective serotonin reuptake inhibitors (SSRIs), nonsteroidal anti-inflammatory drugs (NSAIDs), serotonin norepinephrine reuptake inhibitors (SNRIs), Opioids, anticonvulsants, tricyclic antidepressants (TCAs), Tramadol, and Lithium. Among these, SSRIs and SNRIs jointly contributed 12.6% of the total reported types of poisoning, and exposure to TCAs was low, only 1.9%. The use of more than one substance was common in DSP. In around 47% of cases, multiple substances were ingested. The frequency of intake of benzodiazepines, paracetamol, SNRI, Opioids, TCA, and miscellaneous groups was found significantly different among hospital sites (Table 4). Besides the most frequently used medications or substances for DSP cases, other substances were reported in 916 cases (25.7%). These included about 100 varying classes of toxins/medications. A list of these substances is summarised in Box 1.

## 4. Discussion

This study provides an overview of the characteristics of patients presenting to the ED following DSP in south-eastern suburbs of Melbourne, Victoria, over the last three years. Similar to previous Australian studies [7], we found that women in 18 to 30 years age group were more likely to present following DSP. One important aspect of this study was the inclusion and identification of those presenters who were from a migrant background. In some studies, migrant populations had higher rates of completed suicides [16]. In addition, similar to previous Victorian studies, we found that following DSP, about 50% of the patients presented to all EDs between the late afternoon and midnight [1]. In this study, approximately 30% of the people presenting to our network following DSP were born in countries other than Australia. While this is probably a reflection of the culturally diverse populations located in the area health service [13], we are not aware of any other studies in Australia that have reported this factor in DSP populations. Overseas-born subjects in this study were underrepresented compared to the percentage of those living in the community, particularly in the Dandenong and Monash Medical Centre catchment areas where 60% and 47% of the community, respectively, were born overseas [13]. This finding is notable, and a future research investigation into DSP in culturally sensitive migrant and refugee groups and comparing these with Australian born DSP groups is important to enable more focused and targeted education and preventive measures in the future.

We also assessed socioeconomic status of patients presenting to our ED with DSP by analysing the SEIFA scores of patient address postcodes. Gunnell et al. noted a strong link between socioeconomic deprivation and suicide [17], and SEIFA scores can be utilised to analyse the link between socioeconomic status and illness in Emergency Medicine research in Australia [18]. We were unable to find any current studies utilising SEIFA scores in the analysis of ED presentations for illness in any of the Victorian hospitals. As a result, it was not possible to make a comparison with other areas. However, we did not observe any significant difference in the incidence or epidemiology of DSP between our three hospital EDs despite markedly varying socioeconomic status. We found that DSP patients were more likely to need hospital admission compared to non-DSP patients, with around 40% being admitted to either SSU, a hospital ward, or ICU. The average admission rate for non-DSP patients from our EDs is closer to 30% [15]. It is important to note that around 7% of the patients in this study were admitted from the ED directly to a mental health bed. The higher admission rate for DSP patients compared to non-DSP presentations indicates an increased and potentially preventable burden on the health system [19]. It was beyond the scope of this study to explore the outcome of patients transferred to the mental health or intensive care units or the cost to the health service as a consequence of these admissions [2]. There were differences in the patient disposition categories between EDs and this happened mainly due to the difference in the process of patient disposition (e.g., more patients went from the ED to the short stay unit at Dandenong Hospital). Similarly, transfer of DSP cases to another hospital was high from Casey Hospital due to limited number of specialized facilities such as an Intensive Care Unit. In addition, the factors that related to length of stay in the hospital are also unknown. This finding may be as a result of the differences in the processes and procedures currently occurring between departments. However, examination of these factors was beyond the scope of this study and further investigation is required.

Similar to other Victorian [7] and American studies [20], we found that benzodiazepines were the most frequently ingested drugs. However, we found a difference between the use of paracetamol between our study and those conducted in the UK [20]. Paracetamol was the second most commonly used drug however in this study. However, in the UK, it is the most commonly used substance in DSP [20]. Compared to earlier studies conducted in Australia in the 1990s, we noted a change in the type of poisoning substances ingested. While benzodiazepines and paracetamol still figured highly in many DSP cases, there was an increased incidence of exposure to newer antidepressants such as the SSRIs and SNRIs [12]. There was also a significant reduction in exposure to tricyclic antidepressants compared to older Australian studies (around 10%) [9]. This most likely reflects the change in prescribing patterns for antidepressant and antipsychotic agents and it has been noted in other recent studies done in jurisdictions outside of Victoria [21]. Reasons for this change in the poisoning pattern need further investigations.

## 5. Limitations

- This was a retrospective study utilising a large database with limited details in the information extracted. As a result, only limited interpretations can be made in the results.
- While extreme caution was taken in checking the data, there may have been missed cases due to extreme spelling mistakes in drug names, poisoning substances, and inclusion in poisoning cases in different categories.
- One of the challenges with conducting this study was the inadequacies found in the triage comments. For example, a large variation existed in the spelling of drug names and the use of short hand description of the patients' history and presenting problem.
- Significant difference in the level of significance may be observed due to large sample size of this study, which always may not be clinically significant.
- We were not able to make any major interpretation regarding poisoning severity from this data set. However, with five percent of patients requiring ICU admission, this figure is similar to other DSP studies and indirectly connotes a similar degree of severe poisoning cases as in other studies [22].
- In previous studies, up to 92% of patients presenting to ED with self-harm have an existing psychiatric condition [23, 24]. We were also unable to assess this association due to limited information in the existing database.
- Use of triage comments to define overdose may also be a limitation due to recall bias/unreliable information from the patients or their attendants.

## 6. Conclusions

This is the first study characterising the epidemiology of DSP in South-Eastern Melbourne, one of the fastest growing population areas in Victoria. DSP patients were more likely to be younger females and more likely to require hospital admission compared to non-DSP presentations. While the type of substances used for DSP paralleled those implicated in poisoning in other more contemporary studies, there was a change in the types of substances commonly reported in DSP in earlier Victorian studies. The high incidence of people born outside Australia and lower socioeconomic groups are two areas that require further investigation of the epidemiology of DSP in these vulnerable populations.

## Figures and Tables

**Box 1:**
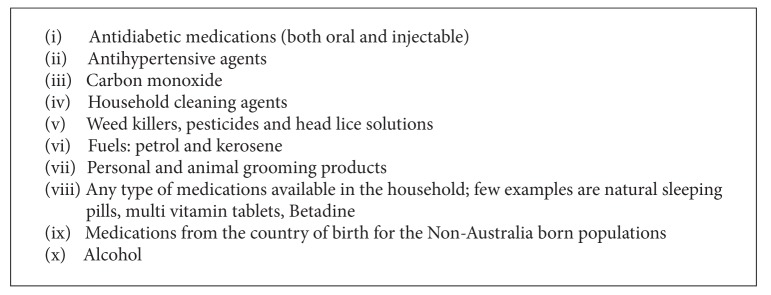
Category of “miscellaneous poisoning substances.”

**Table 1:** Demographics. Age distribution of female patients according to age group (percentage of females of the total number of patients for specific age group is mentioned after the frequency).

	Casey	Dandenong	MMC Clayton	*P* value
Age group	Female	Female	Female	
18–30^*≠*^	249 (68.8)	305 (66.3)	387 (73.3)	0.000*
31–40^*≠*^	147 (51.2)	184 (53.6)	202 (64.5)	0.012*
41–50	167 (65.0)	175 (62.7)	162 (65.6)	0.774
51–60	74 (70.5)	72 (67.9)	60 (69.0)	0.434
>61	27 (58.7)	44 (67.7)	44 (60.3)	0.081
Total	**664 (62.8)**	**780 (62.3)**	**855 (68.5)**	
Marital status				
Single	436 (58.23)	606 (55.60)	763 (65.78)	0.000*
Married/Defacto	329 (37.9)	335 (30.74)	286 (24.66)	0.105
Divorced/separated	78 (8.99)	109 (10.2)	70 (6.09)	0.007*
Widowed	16 (1.84)	21 (1.93)	17 (1.47)	0.678
Not stated/inadequately described	9 (1.04)	19 (1.74)	24 (2.07)	0.035*
Country of birth				
Australia	817 (77.3)	834 (66.56)	908 (72.76)	0.064
Other than Australia^†^	240 (22.7)	419 (33.44)	340 (27.24)	0.000*

*Significant difference exists between hospital areas for the frequency of population (Chi square, *P* < 0.05).

^*≠*^Significant differences exist between hospital areas among age groups, for both sexes. The information for male is not shown in the table.

^†^The major countries in this group were: England, India, New Zealand, Srilanka, Vietnam, and Afghanistan.

**Table 2:** Disposition from Emergency Department.

ED disposition	Casey	Dandenong	MMC Clayton	*P* value
Returning to usual residence	657 (62.22)	357 (28.49)	670 (53.73)	0.000*
Subcategories				
Home	646 (61.17)	349 (27.85)	652 (52.29)	
Mental health residential facility	3 (0.28)	3 (0.24)	10 (0.80)	
Correctional/custodial facility	5 (0.47)	4 (0.32)	2 (0.16)	
Residential care facility/Nursing home	3 (0.28)	1 (0.08)	6 (0.48)	
Ward of the same Hospital Campus Subcategories	271 (25.66)	769 (61.37)	429 (34.40)	0.000*
ED/Short Stay Observation Unit	48 (4.55)	469 (37.43)	34 (2.73)	
Hospital ward	99 (9.38)	113 (9.02)	237 (19.01)	
Mental Health Bed	96 (9.09)	67 (5.35)	88 (7.06)	
ICU	28 (2.65)	120 (9.58)	70 (5.61)	
Left before treatment completed	56 (5.30)	109 (8.70)	104 (8.34)	0.000*
Subcategories				
Left at own risk, without treatment	29 (2.75)	55 (4.39)	58 (4.65)	
Left at own risk, after treatment started	24 (2.27)	49 (3.91)	43 (3.45)	
Left after clinical advice regarding treatment	3 (0.28)	5 (0.40)	3 (0.24)	
Transferred to another Hospital (not Monash Health)	71 (6.72)	13 (1.04)	41 (3.29)	0.000*
CCU	1 (0.09)	5 (0.40)	2 (0.16)	
Deceased	0 (0)	0 (0)	1 (0.08)	

*Significant difference exits between hospital areas (Chi square, *P* < 0.001); for CCU and Deceased thetest was not performed due to small numbers.

**Table 3:** Median length of stay in hospitals.

Department	Median length of stay across all hospitals (minutes)	Median length of stay in individual hospitals (minutes)			
		Casey	Dandenong	MMC Clayton	*P* value
Emergency Department (*n* = 3558)	359.5 (IQR 231–607)	331 (IQR 214–578)	326 (IQR 214–537)	408 (IQR 262–703)	0.0001*
Short Stay Unit (*n* = 550)	376.5 (IQR 182–712)	272 (IQR 141.5–642.5)	394.5 (IQR 187–734.5)	258 (IQR 134–474)	0.0184*

*Significant difference exits between hospital areas (Kruskal Wallis, *P* < 0.05; MMC Clayton was relatively higher).

**Table 4:** Type of poisoning.

Type	Site frequency (percent)			*P* value
	Casey	Dandenong	MMC Clayton	
Benzodiazepines	406 (38.41)	401 (32.00)	496 (39.74)	0.001*
Paracetamol	229 (21.67)	256 (20.43)	305 (24.44)	0.004*
Antipsychotic	124 (11.73)	147 (11.73)	162 (12.98)	0.079
SSRI	101 (9.56)	97 (7.74)	80 (6.41)	0.261
NSAID	95 (8.99)	87 (6.94)	78 (6.25)	0.434
SNRI	101 (9.56)	52 (4.15)	49 (3.93)	0.000*
Opioids	30 (2.84)	54 (4.31)	45 (3.61)	0.033*
Anticonvulsant	18 (1.70)	24 (1.92)	30 (2.40)	0.223
TCA	14 (1.32)	34 (2.71)	19 (1.52)	0.008*
Tramadol	14 (1.32)	26 (2.08)	14 (1.12)	0.069
Lithium	6 (0.57)	8 (0.64)	9 (0.72)	0.738
Miscellaneous groups (substances not included above)	249 (23.56)	369 (29.45)	298 (23.88)	0.000*

*Significant difference between sites (Chi square, *P* < 0.05), number of cases may exceed the total number of patients due to ingestion of more than one poison.
